# The role of gut microbiota in liver metastasis of small cell lung cancer: mechanisms and therapeutic implications

**DOI:** 10.3389/fcimb.2026.1767998

**Published:** 2026-02-18

**Authors:** Yaqiu Xiao, Jiangping Li, Lisha Xiang, Weigang Xiu

**Affiliations:** 1 The Third Hospital of Mianyang, Mianyang, Sichuan, China; 2 Sichuan Mental Health Center, Mianyang, Sichuan, China; 3 The Affiliated Mianyang Hospital of Chongqing Medical University, Mianyang, Sichuan, China; 4 Department of Thoracic Oncology and State Key Laboratory of Biotherapy, Cancer Center, West China Hospital, Sichuan University, Chengdu, China

**Keywords:** gut microbiota, gut-liver axis, immunotherapy resistance, liver metastasis, small cell lung cancer

## Abstract

Small cell lung cancer (SCLC) with liver metastases (LM), represents a highly aggressive clinical challenge characterized by significant morbidity, poor durable responses to chemoimmunotherapy, and limited therapeutic options. While most research has focused on tumor-intrinsic driver mutations and the local liver microenvironment, the remote influence of the gut microbiota on LM-SCLC pathogenesis remains a largely unexplored area. Emerging evidence from other cancer types suggests that the gut microbiota composition and its derived metabolites can modulate systemic immune tolerance, influence hepatic immune surveillance, and affect the efficacy and toxicity of anticancer therapies. This review synthesizes current knowledge on the gut–liver axis in cancer metastasis, with a specific focus on its pathogenesis. We discuss the molecular and immunological pathways through which gut microbial dysbiosis may promote an immunosuppressive liver microenvironment, facilitate the formation of a pro-metastatic niche, and impair anti-tumor responses. Specifically, we detail how translocated microbial products, such as lipopolysaccharide (LPS), and pro-tumorigenic secondary bile acids (SBAs) activate key hepatic immune cells (Kupffer cells, KCs) and stromal cells (hepatic stellate cells, HSCs). This activation modulates key signaling cascades and promotes the survival and outgrowth of circulating SCLC cells. Furthermore, we explore promising microbiota-based therapeutic strategies—including probiotics, prebiotics, fecal microbiota transplantation (FMT), and next-generation microbial therapeutics (NGMTs)—as novel approaches to augment standard-of-care treatments. A deeper understanding of the interplay between the gut microbiota and LM-SCLC is essential for opening new avenues for personalized combination therapies and improving outcomes for this high-risk patient population.

## 1 Introduction

Small cell lung cancer (SCLC) is one of the most refractory malignancies, accounting for approximately 10% to 15% of all lung cancer diagnoses (Rudin et al., 2021). The disease is characterized by its neuroendocrine differentiation, high proliferation kinetics, and the rapid acquisition of therapeutic resistance (Byers and Rudin, 2015). Pathologically, SCLC is defined by near-universal biallelic inactivation of the TP53 and RB1 tumor suppressor genes, resulting in unchecked proliferation and early systemic dissemination (Rudin et al., 2021). Consequently, most patients present with extensive-stage disease (ES-SCLC) at diagnosis (Kim et al., 2025). Although SCLC is initially sensitive to platinum-etoposide chemotherapy combined with immune checkpoint inhibitors (ICIs) such as Atezolizumab or Durvalumab, responses are transient, with rapid relapse typically occurring within 6–9 months (Paz-Ares et al., 2019; Liu et al., 2021a). The median overall survival (OS) for ES-SCLC remains poor at 10–12 months, highlighting an urgent unmet clinical need to identify novel therapeutic targets and resistance mechanisms (Byers and Rudin, 2015). This review posits that resistance and metastatic spread extend beyond tumor-intrinsic genetics to involve external, systemic modulators.

Liver metastasis (LM) is a devastating prognostic factor in ES-SCLC, detected in 30% to 50% of newly diagnosed patients (Fan et al., 2024). The presence of LM is an independent negative prognostic factor correlated with poor survival, lower objective response rates (ORR) to systemic therapy, increased toxicity, and shortened OS (Fan et al., 2024; Kaira et al., 2025). The liver’s high perfusion rate via the portal vein makes it a frequent target for circulating tumor cells (CTCs) (Ohtani and Hara, 2021). For SCLC cells to colonize the liver, they must overcome the organ’s innate immune surveillance and adapt to its unique microenvironment (Massagué and Obenauf, 2016). The high frequency and poor outcomes associated with LM suggest that the liver, rather than acting as an immune barrier, may be pre-conditioned to accept SCLC cells, a state often termed the “fertile soil” (Kaplan et al., 2005; Massagué and Obenauf, 2016). Understanding this pre-conditioning process, particularly the contribution of remote factors, is paramount for developing effective, LM-specific interventions.

The classical perspective on metastasis emphasized tumor cell characteristics and the local microenvironment (Massagué and Obenauf, 2016). However, the limited efficacy of therapies based on this paradigm has necessitated a shift toward a systemic view of cancer progression, one that prominently features the gut-liver axis as a non-tumor-intrinsic determinant of metastatic risk, immune tolerance, and therapeutic outcome (Yu and Schwabe, 2017; Zheng and Wang, 2021). This anatomical and functional relationship is established by the portal vein, which links the microbial-rich intestinal lumen to the liver parenchyma (Ohtani and Kawada, 2019). The gut microbiota functions as a metabolic and immunological organ, sending a continuous stream of modulatory signals—including short-chain fatty acids (SCFAs), microbial products, and host-modified metabolites—to the liver (Ohtani and Hara, 2021). Dysbiosis, an imbalance of this microbial ecosystem, can remotely trigger chronic hepatic inflammation, metabolic dysregulation, and immunosuppression, thereby altering the liver’s capacity for anti-tumor surveillance against circulating SCLC cells (Deepika et al., 2025). This review details how dysbiosis may create a permissive hepatic microenvironment for SCLC and drive chemo-immunotherapy resistance, providing a rationale for microbial modulation in LM-SCLC.

This review integrates current knowledge on the impact of the gut microbiota on liver metastasis, applying these insights to the understudied pathology of LM-SCLC (Qi et al., 2020).

## 2 The gut microbiome and cancer

The human gut harbors a complex ecosystem of microorganisms, dominated by bacterial phyla such as Firmicutes and Bacteroidetes (Wang et al., 2018). This microbial community engages in a symbiotic relationship with the host, performing vital functions extending beyond mere nutrient absorption.

### 2.1 Microbial composition, core functions, and the pathological state of dysbiosis

A healthy, diverse microbiota is essential for host physiology: it ferments non-digestible carbohydrates, synthesizes micronutrients, recycles host molecules, and confers colonization resistance against pathogens (Wu and Wu, 2012). Dysbiosis is a persistent imbalance characterized by reduced microbial diversity and a compositional shift toward pro-inflammatory pathobionts and away from protective commensals (Kulecka et al., 2024). This state is a foundational trigger for numerous pathologies, including chronic inflammation, metabolic disorders, and cancer progression, by altering the systemic immune “set point” (Jia et al., 2008; Zhang et al., 2023).

### 2.2 Core regulatory mechanisms in cancer progression

The gut-associated lymphoid tissue (GALT) is one of the largest immune compartments, where immune cells are continuously trained by microbial antigens and metabolites (Pačes et al., 2025). This interaction shapes the peripheral T cell repertoire and the function of adaptive and innate immune cells. The microbiome dictates the balance between a robust anti-tumor response and an immunosuppressive state (Helmink et al., 2019). Dysbiosis is often associated with systemic, low-grade inflammation that paradoxically promotes tumor growth by activating immunosuppressive cells and inducing chronic tissue damage, a prerequisite for metastatic niche formation (Zhao et al., 2023). Microbial metabolism generates unique small-molecule compounds that enter systemic circulation and act as signaling molecules on distant cells, including those in the liver (Yang et al., 2023).

Fermentation of dietary fiber by anaerobes yields Short-Chain Fatty Acids (SCFAs) — primarily acetate, propionate, and butyrate (Dora et al., 2024). Butyrate is a critical energy source for colonocytes and, systemically, functions as a histone deacetylases (HDAC) inhibitor, epigenetically modulating host cell proliferation and apoptosis (Fung et al., 2012). Loss of SCFA-producing bacteria removes this protective signal, leading to barrier dysfunction, whereas adequate SCFA levels may suppress pro-metastatic inflammation (Liu et al., 2018). SCFAs primarily signal through G-protein coupled receptors GPR41 and GPR43 on immune cells. Furthermore, the microbiota plays a rate-limiting role in L-Tryptophan metabolism, generating indole derivatives that act as ligands for the Aryl Hydrocarbon Receptor (AhR) (Gao et al., 2018; Platten et al., 2019). AhR signaling regulates Treg differentiation, promotes tight junction protein expression, and induces IL-22 production, thereby maintaining intestinal homeostasis (Kang et al., 2025). Dysbiosis can shift Tryptophan metabolism toward the kynurenine pathway, which promotes immunosuppression and Treg expansion, a state relevant to SCLC pathogenesis and ICI resistance (Zhai et al., 2015; Platten et al., 2019; Dora et al., 2020).

### 2.3 Intestinal barrier integrity and systemic translocation

The intestinal epithelial layer, maintained by tight junction proteins, forms a selective barrier (Ulluwishewa et al., 2011). Dysbiosis can trigger the breakdown of these junctions, a condition known as increased intestinal permeability or “leaky gut” (Saggioro, 2014). This breach allows the uncontrolled systemic translocation of immunogenic microbial products—such as LPS, peptidoglycans (PAMPs), and bacterial DNA—directly into the portal circulation (Rooks and Garrett, 2016; Vivarelli et al., 2019). This continuous influx of microbial components into the liver is a primary trigger for the chronic hepatic inflammation and immunosuppression that facilitate SCLC metastasis.

The microbiome’s clinical relevance is strikingly demonstrated by its role in modulating responses to anticancer therapies. Clinical data in melanoma and non-small cell lung cancer (NSCLC) shows that patients with a favorable microbial composition exhibit significantly higher ORR and improved survival following PD-1/PD-L1 blockade (Routy et al., 2018). These commensals are thought to enhance CD8+ T cell priming and trafficking into the tumor site (Sivan et al., 2015). Conversely, antibiotic exposure or an unfavorable microbial profile is linked to resistance to immune checkpoint inhibitors (ICIs) (Nyein et al., 2022). Given the low and transient ICI response rates in LM-SCLC (Paz-Ares et al., 2022), it is plausible that gut dysbiosis contributes to this resistance by fostering an “immune-cold” and immunosuppressive liver microenvironment.

## 3 Anatomical and physiological basis of the gut-liver axis

The liver’s function as a metabolic hub and immunological filter is linked to its anatomical relationship with the gastrointestinal tract, termed the gut-liver axis. The portal vein system is a central component of the liver’s unique vascular architecture (Ohtani and Kawada, 2019). The portal vein collects nutrient-rich blood, metabolites, and gut-derived components from the gastrointestinal tract and directs this “first pass” flow into the liver sinusoids (Ohtani and Kawada, 2019). This design ensures the liver is the first organ exposed to microbial-derived signals such as LPS, Secondary Bile Acids (SBAs), and SCFAs (Visekruna and Luu, 2021). Pathological intestinal permeability (leaky gut) directly translates to a chronic increase in the flux of these microbial components, leading to a state of chronic hepatic inflammation (Kubes and Jenne, 2018).

The liver is uniquely adapted to maintain immune tolerance toward the constant, low-level flow of non-pathogenic gut antigens, preventing chronic hepatitis (Shetty et al., 2018). This baseline tolerance is maintained by specialized non-parenchymal immune cells (Kubes and Jenne, 2018). As liver-resident macrophages located in the sinusoids, Kupffer cells (KCs) serve as the primary gatekeepers. They clear circulating bacteria and LPS via pattern recognition receptors (PRRs) without triggering an aggressive inflammatory response (Dixon et al., 2013). Liver Sinusoidal Endothelial Cells (LSECs) promote hepatic tolerance by presenting antigens to T cells without co-stimulation, which leads to T cell anergy or deletion (Shetty et al., 2018). However, when chronic microbial stress from dysbiosis overwhelms this homeostatic mechanism, the hepatic microenvironment shifts. The resulting low-grade inflammation initiates liver damage and creates an immunosuppressive and pro-fibrotic environment (Zheng and Wang, 2021), transforming the liver from an immune filter into a “fertile soil” that supports the engraftment and outgrowth of circulating SCLC cells (Kaplan et al., 2005).

## 4 Potential mechanisms of the gut microbiome in SCLC liver metastasis

**Figure 1:**
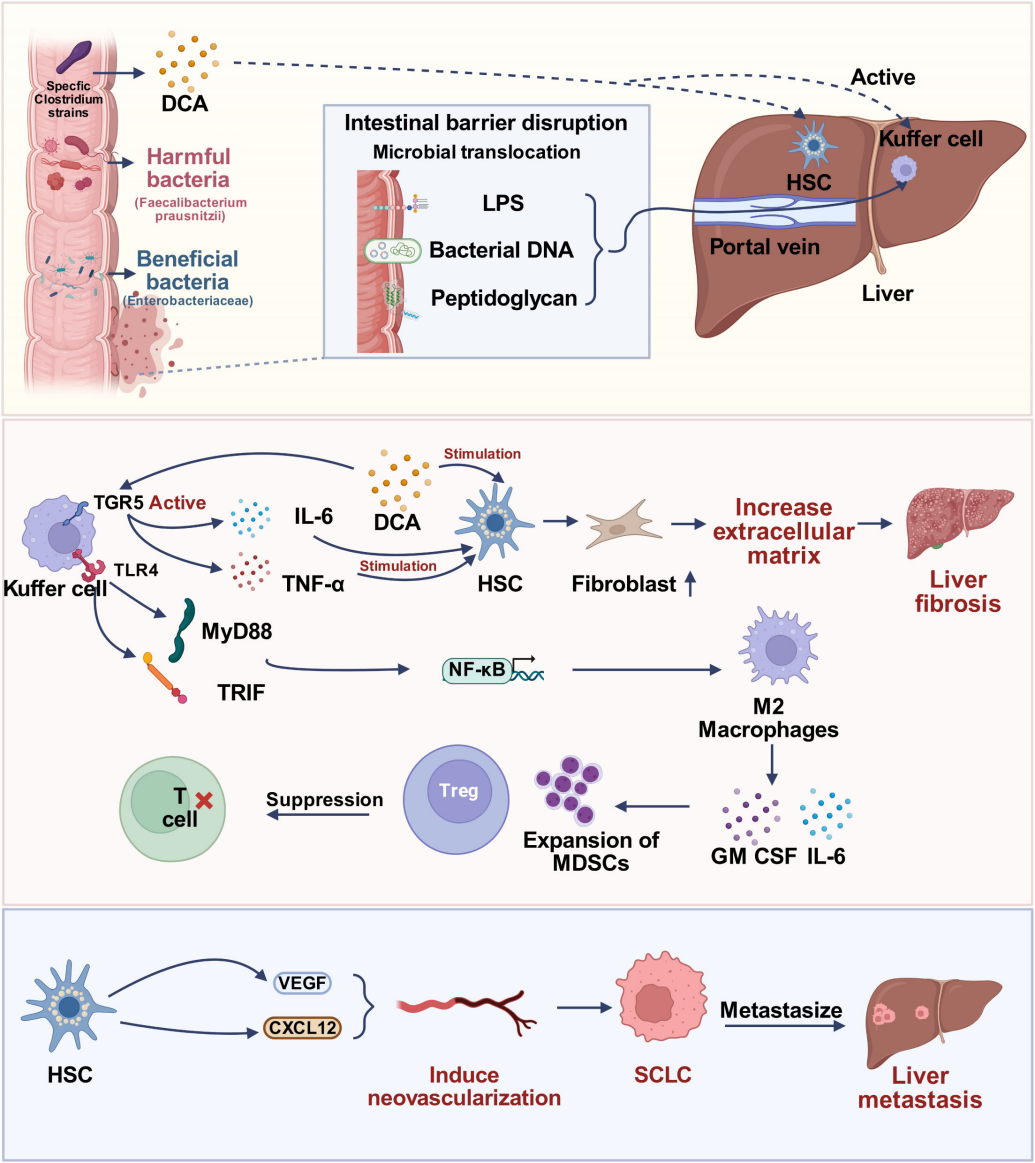
Proposed mechanisms by which the gut microbiome promotes liver metastasis in small cell lung cancer (SCLC). Gut dysbiosis disrupts the intestinal barrier, facilitating the translocation of microbial products such as lipopolysaccharide (LPS) and increasing the production of deoxycholic acid (DCA). In the liver, LPS activates Kupffer cells (KCs) via TLR4, fostering an immunosuppressive microenvironment through the recruitment of myeloid-derived suppressor cells (MDSCs) and regulatory T cells (Tregs). Concurrently, DCA activates hepatic stellate cells (HSCs), resulting in extracellular matrix deposition and hepatic fibrosis. Together, these processes establish a fibrotic and pro-angiogenic niche that enables colonization and metastasis of SCLC cells.

The gut microbiome facilitates LM-SCLC by promoting systemic immunosuppression and conditioning the hepatic pre-metastatic niche (Sater et al., 2022; Liu et al., 2023). The influx of translocated microbial products drives immune cell dysfunction within the liver, neutralizing anti-tumor surveillance (**Figure 1**).

A central event is the chronic activation of Kupffer cells (KCs). Sustained LPS translocation via the portal vein facilitates LPS binding to the Toll-like receptor 4 (TLR4) complex on the KC surface (Seki and Schwabe, 2015). This initiates robust activation of MyD88-dependent and TRIF-dependent pathways, leading to the nuclear translocation of NF-κB (Seki and Schwabe, 2015). This sustained, NF-κB-mediated signal drives the polarization of KCs towards an immunosuppressive, M2-like Tumor-Associated Macrophages(TAMs) (Tacke, 2017; van der Heide et al., 2019).

Gut dysbiosis remotely promotes the expansion of Myeloid-Derived Suppressor Cells (MDSCs) in the bone marrow, driven by microbial-induced systemic IL-6 and GM-CSF signaling (Gabrilovich, 2017; Krishnamoorthy et al., 2021; Lu et al., 2024). Upon exposure to gut-derived LPS, Kupffer cells are activated via the TLR4 signaling cascade. This interaction triggers the phosphorylation and nuclear translocation of NF-κB, a pivotal transcription factor that upregulates the expression of pro-inflammatory chemokines and cytokines (Bartolini et al., 2021; Wang et al., 2025). Specifically, activated KCs secrete high levels of C-C motif chemokine ligand 2 (CCL2) and granulocyte-macrophage colony-stimulating factor (GM-CSF) (Sierra-Filardi et al., 2014; Fei et al., 2021). CCL2 functions as a potent chemoattractant by binding to its cognate receptor, CCR2, expressed on the surface of circulating MDSCs, thereby driving their directional migration from the bone marrow and peripheral blood into the hepatic tumor microenvironment (Li et al., 2022). Concurrently, GM-CSF binds to the GM-CSF receptor on MDSCs, which not only facilitates their recruitment but also enhances their survival and immunosuppressive activity. This coordinated signaling network establishes a local immunosuppressive niche that protects metastatic cells from cytotoxic T-cell surveillance (Zhu et al., 2024a). Polymorphonuclear MDSCs (PMN-MDSCs) are abundant in cancer and suppress T cells primarily through the high-level generation of Reactive Oxygen Species (ROS) and Nitric Oxide (NO) via inducible Nitric Oxide Synthase (iNOS) (Bronte et al., 2016; Tacke, 2017). Monocytic MDSCs (M-MDSCs) can differentiate into suppressive TAMs. M-MDSCs suppress T cells by upregulating Arginase-1 (ARG1), which depletes L-arginine from the microenvironment, leading to T cell anergy (Bronte et al., 2016).

Simultaneously, microbial metabolites reinforce the Regulatory T Cell (Treg) population. Tryptophan catabolites generate ligands that promote AhR activation (Wheeler et al., 2017), which drives the *de novo* differentiation and stabilization of Foxp3+ Tregs (Ha, 2009; Platten et al., 2019). These Tregs accumulate in the liver, where they neutralize effector T cell responses against SCLC antigens (Wang et al., 2012).

The gut microbiome also promotes metastasis by physically and metabolically conditioning the liver tissue, a process known as pre-metastatic niche formation (Kaplan et al., 2005). Gut bacteria are essential for transforming primary bile acids (PBAs) into Secondary Bile Acids (SBAs), such as Deoxycholic Acid (DCA) and Lithocholic Acid (LCA) (Ramírez-Pérez et al., 2018). Dysbiosis, particularly the overgrowth of specific Clostridium strains, can result in a pathologically high concentration of oncogenic DCA in the portal circulation (Song et al., 2024). The transformation of primary bile acids into SBAs such as DCA and LCA is not governed by a single species but relies on a coordinated multi-step enzymatic network involving diverse microbial taxa. The process initiates with the deconjugation of glycine- or taurine-conjugated primary bile acids, a ‘gateway’ reaction catalyzed by bile salt hydrolase (BSH) enzymes. This activity is widely distributed among the gut microbiota, with high BSH activity identified in genera from the Bacteroidaceae and Actinomycetaceae families (Ridlon et al., 2006; Jones et al., 2008). The resulting unconjugated bile acids then serve as substrates for 7-dehydroxylation, a specialized enzymatic reaction primarily restricted to a small subset of bacteria, most notably Clostridium species from cluster XIVa (Wahlström et al., 2016). Thus, the accumulation of carcinogenic SBAs in the liver microenvironment is the net result of a synergistic regulatory chain where BSH-rich genera facilitate substrate availability for 7-dehydroxylation species.

DCA activates Hepatic Stellate Cells (HSCs) (Saga et al., 2018), the primary source of fibrotic tissue. Upon activation, HSCs trans-differentiate into myofibroblasts and increase the deposition of the Extracellular Matrix (ECM) (Drabsch and Ten Dijke, 2012). This creates a rigid and growth-factor-rich pre-metastatic niche (Kaplan et al., 2005; Drabsch and Ten Dijke, 2012). DCA activates the TGR5 bile acid receptor on KCs (Kawamata et al., 2003), leading to the release of IL-6 and TNF-α, which indirectly stimulates HSCs. DCA also activates the Farnesoid X Receptor (FXR) in HSCs, promoting the transcription of pro-fibrotic genes (Ding et al., 2024). Conversely, beneficial SCFAs like butyrate can inhibit HSC activation *in vitro* by modulating HDAC activity, suggesting a healthy microbiota opposes fibrotic niche development (Visekruna and Luu, 2021).

This sustained microbial-driven inflammation leads to progressive hepatic fibrosis (Guo and Teng, 2015; Zhang et al., 2021). This fibrotic microenvironment is conducive to tumor cell colonization (Heinz et al., 2022). The increased tissue stiffness alters cellular mechanosensing. This rigidity promotes SCLC cell survival and proliferation through the activation of the YAP/TAZ mechanotransduction pathway, which confers stemness and resistance to apoptosis (Guo and Teng, 2015). Activated HSCs secrete Vascular Endothelial Growth Factor(VEGF), Fibroblast Growth Factor(FGF) (Zhu et al., 2020) and CXCL12, which facilitate angiogenesis and guide circulating SCLC cells to the pre-metastatic site (Pei et al., 2023). Thus, dysbiosis effectively creates a structurally compromised and fibrotic rigid liver primed for SCLC survival.

The gut microbiome modulates the efficacy and toxicity of systemic anticancer treatments. An unfavorable microbial composition is a potent determinant of primary ICI resistance by promoting T cell anergy and fostering an “immune-cold” phenotype (Liu et al., 2021b; Yang et al., 2023). Microbial-mediated metabolic pathways, such as kynurenine catabolis, contribute to CD8+ T cell exhaustion, characterized by co-expression of multiple inhibitory receptors (Wirthgen et al., 2018). The absence of key immune-potentiating bacteria such as Bifidobacterium and Akkermansia means the systemic immune circuits required for a successful ICI response are not fully activated (Sivan et al., 2015). This may explain the low and transient ICI responses observed in LM-SCLC.

The gut microbiota can also influence the therapeutic window of chemotherapeutic agents, termed the “chemo-microbiome axis” (Alexander et al., 2017). Specific gut microbes express high levels of β-glucuronidase (Nakamura et al., 2002). This enzyme reverses the liver’s detoxification by hydrolyzing the inactive irinotecan metabolite (SN-38G) back into its highly toxic, active form (SN-38) in the intestinal lumen (Hsieh et al., 2015). This microbial reactivation leads to severe, dose-limiting toxicities, often necessitating dose reduction (Yin et al., 2022). This interaction can result in unpredictable drug bioavailability at the liver metastasis site, contributing to therapeutic failure. Furthermore, the microbiota can modulate host drug metabolism by regulating hepatic CYP enzymes via AhR and FXR signaling (Liu et al., 2022).

## 5 Therapeutic strategies targeting the gut microbiome

Targeted modulation of the gut microbiome offers a novel approach to overcome therapeutic resistance and improve outcomes in LM-SCLC (Xia et al., 2025). **Table 1** shows the cohort size, specific changes in microbial abundance, and their statistical associations with clinical outcomes.

**Table 1:** Key studies linking gut microbiota dysbiosis, metabolites, and therapeutic outcomes relevant to the SCLC liver metastasis axis.

Study context & design	Comparison group	Key microbial/metabolite alterations	Quantitative clinical/experimental outcomes	Relevance to SCLC-LM pathogenesis	Reference
A. Clinical Evidence					
SCLC Immunotherapy Cohort (Retrospective, *n* = 49)	R Group *vs.* NR Group	Diversity: Desulfobacterota was significantly enriched in the R group (*p* < 0.05).	Beta Diversity revealed a significant separation between the R and NR groups following treatment (*p* < 0.05)	Establishes that gut diversity and specific taxa are prognostic markers for ICI efficacy in SCLC.	(Sun et al., 2024)
Lung Cancer & Antibiotics (Retrospective, *n* = 2028)	ATB Users *vs.* Non-Users	Dysbiosis: ATB induced depletion of commensals.	Median OS: 10 months (ATB+) *vs.* 15 months (ATB-), HR 1.50, *p* = 0.00014.	Quantifies the severe impact of dysbiosis on survival, relevant to fragile SCLC-LM patients.	(Elkrief et al., 2024)
B. Mechanisms of Gut-Liver Axis					
Liver Metastasis Model (Murine model)	Liver Metastasis *vs.* Primary Tumor	Translocation: Live Fusobacterium detected in hepatic metastases. Persistence: Bacteria survive in metastatic niche.	Tumor Load: Metronidazole treatment reduced liver metastatic burden (*p* < 0.01).	Proves that gut bacteria can physically translocate to the liver and promote metastatic growth.	(Bullman et al., 2017)
Metabolite-Driven Inflammation (Mechanistic model)	High-Fat Diet/Liver Injury	Metabolite: Deoxycholic Acid (DCA). Target: Hepatic Stellate Cells (HSCs).	Threshold: Higher DCA concentrations trigger DNA damage and SASP phenotype in HSCs.	Provides the quantitative threshold for secondary bile acids to create a pro-metastatic liver niche.	(Yoshimoto et al., 2013)
LPS-TLR4 Signaling (Mice model)	TLR4+ *vs*. TLR4- cells	Signal: LPS-TLR4 axis activation. Effect: Up-regulation of CCL2/PD-L1.	LPS activate immunosuppressive signaling in hepatic myeloid cells.	Defines the metabolite concentration required to activate the Kupffer cell-MDSC axis described in the review.	(Ochi et al., 2012)
C. Therapeutic Intervention					
FMT & Immunotherapy (Translational Model)	Mice + Responder FMT *vs.* Mice + Non-Responder FMT	Taxa: Akkermansia muciniphila abundance. Immune: CD8+/Treg ratio in tumor bed.	Tumor Control: Responder-FMT significantly delayed tumor growth (*p* < 0.05). Mechanism: Increased IFN-γ production.	Validates FMT as a strategy to transfer “responder” phenotypes, supporting its use in refractory SCLC-LM.	(Routy et al., 2018)

SCLC, Small cell lung cancer; LM, liver metastases; R, Responders; NR, Non-Responders; ATB, Antibiotic; OS, Overall survival; DCA, Deoxycholic acid; HSCs, Hepatic Stellate Cells; SASP, senescence-associated secretory phenotype; LPS, Lipopolysaccharide; TLR4, Toll-like receptor 4; MDSCs, Myeloid-Derived Suppressor Cells; FMT, Fecal microbiota transplantation.

Probiotics aim to introduce key immune-potentiating species. Strains such as Bifidobacterium (Sivan et al., 2015) and Akkermansia muciniphila (Zhu et al., 2024b) are reported to enhance T cell responses. Specific strains may also restore intestinal barrier integrity via AhR activation (Stockinger et al., 2021), thereby decreasing LPS translocation. Prebiotics (Holscher, 2017) function as nourishment for endogenous beneficial bacteria, promoting robust SCFA production (Peng et al., 2007). This increased SCFA pool exerts systemic anti-inflammatory effects and may oppose HSC activation in the liver (Mijangos-Trejo et al., 2023). Synbiotics, which combine a probiotic with its preferred prebiotic substrate, aim for a synergistic effect (Cortez-Pinto et al., 2016).

Diet is also a powerful tool for modifying the gut microbiome. A “pro-efficacy” diet, characterized by high-fiber, whole-food consumption, promotes the growth of SCFA-producing bacteria (Bach Knudsen et al., 2018). It is critical to discourage the Western-style diet—characterized by high saturated fat, high sugar, and low fiber—as it is linked to dysbiosis, LPS leakage, and chronic inflammation, which collectively fuel the pre-metastatic niche (Singh et al., 2017).

FMT is the most comprehensive method for restructuring a dysbiosis ecosystem by transplanting a functional microbial community from a healthy donor. FMT has successfully reversed ICI resistance in patients with advanced melanoma and NSCLC by reintroducing key immune-potentiating strains (Baruch et al., 2021; Duttagupta et al., 2025). To maximize therapeutic gain, donors should be selected based on the ‘Responder-FMT’ principle—utilizing stool from SCLC patients who have achieved a durable objective response to chemo-immunotherapy. Alternatively, ‘super-donors’ should be screened for high alpha-diversity and for enrichment in bacteria of the Ruminococcaceae and Lachnospiraceae families (He et al., 2021). While liver metastasis can compromise hepatic function, emerging consensus indicates that FMT is safe in patients with chronic liver disease provided specific exclusion criteria are met (Zhou et al., 2025). Clinical trials (NCT05502913) suggest that FMT is most effective when administered as a priming therapy 7–14 days prior to the first cycle of immunotherapy, or concurrently with the first dose, followed by maintenance boosters every 3–4 weeks to ensure engraftment. For LM-SCLC, FMT holds potential to restore microbial diversity, strengthen barrier function, and re-sensitize the hepatic tumor microenvironment to immunotherapy.

Due to risks associated with crude FMT, the field is moving toward Next-Generation Microbial Therapeutics (NGMTs) (Abouelela and Helmy, 2024). These consist of defined, rationally selected bacterial consortia. NGMTs offer superior safety, scalability, and reproducibility. Future ‘designer’ probiotics (Bai et al., 2023) may be genetically engineered to deliver specific therapeutic payloads locally to the mucosal surface, enhancing ICI efficacy while reducing systemic toxicity (Goswami et al., 2024). The clinical translation of NGMTs relies on precise strain selection criteria derived from responder profiling. Preclinical models of lung cancer have established Akkermansia muciniphila as a critical efficacy indicator; its abundance positively correlates with elevated interferon- (IFN-) production and CD8+ T-cell infiltration in the liver tumor microenvironment (Derosa et al., 2022). Recent preclinical data further suggests that specific bacterial metabolites, such as the novel molecule Bac429 or short-chain fatty acids (SCFAs), can be used as biomarkers to screen candidate strains for their ability to convert ‘cold’ tumors into immunologically ‘hot’ phenotypes (Newsome et al., 2026). Although clinical evidence for NGMTs in LM-SCLC remains limited, NGMTs may offer a safety advantage over whole-stool transplants by eliminating the risk of transferring multidrug-resistant pathobionts.

The negative impact of non-strategic antibiotic (ABX) use on ICI efficacy is well-documented (Hobeika et al., 2024). ABX causes microbial depletion and is associated with diminished ORR and reduced survival, particularly when administered near the start of immunotherapy (Pinato et al., 2019). Future clinical guidelines should prioritize narrow-spectrum agents to preserve microbial diversity (Guerrero et al., 2025). For patients receiving Irinotecan, specific β-glucuronidase inhibitors can prevent the microbial reactivation of SN-38 (Hsieh et al., 2015), disarming the microbial contribution to toxicity without broad-spectrum ABX. Prophylactic use of protective probiotics or prebiotics may be warranted to maintain critical microbial functionality and reduce chemotherapy-induced gastrointestinal toxicity (Śliżewska et al., 2020).

## 6 Challenges and future directions in LM-SCLC research

Translating microbiome modulation strategies into clinical practice for LM-SCLC faces significant challenges. A fundamental hurdle is moving from observational correlation to establishing causal mechanisms (Long et al., 2023). Future research must integrate deep metagenomic, metatranscriptomics, and metabolomic profiling (Xue et al., 2023; Zeriouh et al., 2023) to define the functional output of the microbiome.

Causal roles must be validated using gnotobiotic mouse models colonized with specific human microbial consortia or single organisms to confirm that a species or metabolite is necessary and sufficient to drive LM-SCLC pathogenesis (Ashique et al., 2024). Concerted efforts are needed to identify microbes uniquely associated with LM-SCLC. A crucial direction is investigating how the gut-liver axis influences SCLC lineage plasticity—the transformation of sensitive SCLC-A subtypes to resistant SCLC-P or SCLC-N subtypes (Raso et al., 2021)—in the hepatic metastatic environment.

The heterogeneity of the human microbiome poses a challenge for developing standardized clinical tools. The goal is to identify robust microbial and metabolic biomarkers (Oh et al., 2021) for patient management. Such biomarkers should ideally forecast which SCLC patients are at high risk for LM or ICI resistance. The field must prioritize functional markers—such as quantifiable ratios of key metabolites in peripheral blood—over taxonomic abundance, as functional outputs are often more stable and reproducible (Marchesi et al., 2016).

The therapeutic landscape is moving from crude interventions to precision engineering. A key goal is developing rationally selected microbial consortia (Fan and Pedersen, 2021) customized to correct specific metabolic or immunological deficits in LM-SCLC patients. Live biotherapeutic products (LBPs) face immense regulatory hurdles, including the need for cGMP-compliant manufacturing and robust quality control (O’Toole et al., 2017). Developing small-molecule inhibitors that target key microbial enzymes without affecting the entire commensal community is a promising strategy.

Successful translation requires rigorous, rational, and well-controlled clinical trials in the high-risk LM-SCLC population. Future trials should consider utilizing adaptive designs to rapidly screen different NGMT compositions or FMT donors for their ability to augment ICI efficacy in SCLC (Dronkers et al., 2020; Vandeputte, 2020). These trials must also implement robust protocols for the standardized longitudinal collection of multiple biospecimens at critical time points. Trials must integrate sophisticated correlative endpoints, such as changes in hepatic MDSC/Treg ratios, circulating DCA levels, and T cell activation markers, as functional readouts of the intervention’s biological impact.

The focus must be on combination trials to prove that microbial intervention augments the efficacy of standard-of-care chemo-immunotherapy, demonstrating a durable improvement in survival (Takada et al., 2021).

## 7 Conclusion

The gut-liver axis, and the microbial dysbiosis that defines its pathological state, constitutes a potent, underappreciated, and targetable pathway driving the aggressive nature of SCLC liver metastasis (Wang et al., 2021). Microbial alterations—mediated through intestinal barrier compromise, systemic translocation of inflammatory products, and profound metabolic reprogramming—promote an immunosuppressive hepatic microenvironment and accelerate pro-metastatic niche formation (Kaplan et al., 2005; Liu et al., 2023). This chronic conditioning of the “fertile soil” is a critical, non-tumor-intrinsic factor contributing to primary ICI resistance and therapeutic failure (Massagué and Obenauf, 2016). Targeted strategies, including FMT, precision NGMTs, and engineered probiotics (Abouelela and Helmy, 2024), offer a novel therapeutic avenue to remodel this tumor-supportive environment, mitigate treatment-related toxicities, and augment the efficacy of standard-of-care therapies (Abouelela and Helmy, 2024; Goswami et al., 2024). By rigorously investigating the molecular mechanisms of the gut-liver axis and leveraging therapeutic microbial modulation, future translational research holds the potential to fundamentally transform the prognosis for this difficult-to-treat patient population.
